# Evaluating the Impact of a Health Literacy Intervention on the Quality of Dietetic Communication in Outpatient Kidney Care

**DOI:** 10.1111/jhn.70296

**Published:** 2026-06-25

**Authors:** Jenna Mazabow, Denelle Cosier, Kelly Lambert

**Affiliations:** ^1^ School of Medical, Indigenous and Health Sciences University of Wollongong Wollongong New South Wales Australia; ^2^ XS Network Tech Pty Ltd Sydney Australia; ^3^ Department of Nutrition and Dietetics Illawarra Shoalhaven Local Health District Wollongong New South Wales Australia

**Keywords:** communication, DIET‐COMMS, dietitian, health literacy, teachback

## Abstract

**Introduction:**

Effective communication between patients and health professionals is essential in chronic disease management. In the context of conditions like chronic kidney disease this is especially critical given the complexity of dietary advice that may be required. The technical quality of dietetic communication in nephrology settings remains underexplored. This study evaluated the technical quality of dietetic communication in renal outpatient clinics and assessed changes in communication following implementation of a health literacy intervention.

**Methods:**

This secondary analysis utilised audio recordings from a quasi‐experimental pre‐post study conducted in three outpatient kidney clinics. The health literacy sensitive intervention included teachback, use of a renal diet‐specific question prompt sheet (QPS), and plain English dietary education resources. Paired consultations (initial and review) were assessed using the validated DIET‐COMMS tool with scores converted to percentages and classified as poor (< 40%), average (41%–75%), or good (> 75%).

**Results:**

Fifty‐four appointments from 27 participants were analysed. The mean DIET‐COMMS score was 80.9% (SD 10.2), with 81.5% of consultations demonstrating high‐quality communication. No significant changes in DIET‐COMMS scores were observed between pre‐ and post‐intervention groups, appointment types, or dietitian experience levels. However, appointments using both teachback and QPS achieved the highest mean scores (86.0%). Notably, active listening and interview structure scores declined post‐intervention, while provision of condition‐specific dietary information improved.

**Discussion:**

The intervention did not result in further increases in overall communication scores from baseline but influenced specific communication behaviours. The decline in active listening may reflect increased cognitive load or altered consultation flow due to prompt sheet use. Combined use of teachback and the QPS shows promise for enhancing communication quality.

**Conclusion:**

Dietitians in nephrology outpatient settings generally demonstrate high‐quality communication. Targeted interventions can influence specific communication behaviours, highlighting the need for ongoing training and structured tools to support effective, patient‐centred care.

## Summary

- Dietitians in nephrology outpatient settings demonstrated high‐quality communication, with over 80% of consultations meeting criteria for effective practice.
- Integration of health literacy sensitive communication techniques such as teachback and question prompt sheets may enhance communication quality, though implementation may disrupt consultation flow.
- Targeted communication skills training would be beneficial to support dietitians to maintain high‐quality, patient‐centred communication, especially when integrating new communication tools or interventions.

## 1 Introduction

Effective communication is a cornerstone of quality healthcare [1]. This is particularly important in the management of chronic conditions such as chronic kidney disease (CKD), where dietary interventions are complex and require sustained patient engagement [2]. Dietitians play a critical role in translating the science of nutrition into actionable advice, yet the quality of their communication skills and how these skills influence patient outcomes remains underexplored in nephrology settings. While communication training is recognised as essential in dietetic education [3], there is limited empirical evidence evaluating the technical quality of dietetic communication in real‐world clinical practice, especially in the context of CKD.

CKD presents unique challenges for dietary management. Patients must navigate intricate nutrient restrictions involving fluid, sodium, potassium, phosphorus and protein, often while managing multiple comorbidities and fluctuating clinical parameters. The complexity of this information, coupled with the emotional burden of chronic illness, underscores the need for dietitians to employ clear, empathetic and patient‐centred communication strategies. Health literacy sensitive communication approaches such as using plain language, teachback techniques and question prompt sheets (QPS) have shown promise in improving patient understanding and engagement [4]. However, the extent to which dietitians integrate these strategies into routine practice, and how their communication skills are impacted following targeted interventions, remains unclear.

Previous research has demonstrated that communication skills training can enhance practitioner confidence [5] and patient satisfaction [6], yet few studies have rigorously assessed the technical quality of dietetic communication using validated tools. There is limited quantitative evidence of interventions to improve communication quality, despite communication being central to delivering complex dietary advice. The DIET‐COMMS tool [7], developed specifically for dietetic consultations, offers a structured framework for evaluating communication behaviours across multiple domains, including information exchange, rapport building and patient engagement. It has been validated in various settings [7, 8] and provides a reliable method for assessing both the presence and quality of key communication elements [7]. Despite its utility, the DIET‐COMMS tool has not been widely applied in nephrology outpatient clinics, nor has it been used to evaluate the impact of communication‐focused interventions in this context.

To our knowledge, only one study [4] has explored how the quality of dietetic communication skills may impact outcomes in patients with CKD. Given the importance of effective communication in delivering complex health information and supporting behaviour change, further investigation into dietetic communication in nephrology is warranted. This study addresses this gap by evaluating the technical quality of dietitian communication intervention in outpatient kidney clinics and examining changes following a targeted intervention.

The intervention, implemented as part of the HERALD study [4], incorporated health literacy‐sensitive strategies including teachback, a renal diet QPS, and the use of plain English education resources. Audio recordings of dietetic consultations were collected pre‐ and post‐intervention, providing a rich dataset for secondary analysis. The primary aim of this study was therefore to evaluate the technical quality of communication skills demonstrated by dietitians in nephrology outpatient settings. A secondary aim was to explore which components of dietetic communication changed following participation in the communication intervention.

## 2 Materials and Methods

This study is a secondary analysis of audio recordings from a quasi‐experimental pre‐post study that evaluated the impact of a health literacy‐sensitive model of dietetic care in three kidney care outpatient clinics in one regional Australian health district [4]. The aim of the study was to determine if an intervention focused on improved communication including teachback, a renal diet QPS [9] and use of plain English education resources as key components of the intervention would result in improved diet quality or quality of life in patients with kidney disease. The key findings from this work were lower fail to attend rates in the revised model of care, and improvements in fruit (*p* = 0.03) and vegetable intake (*p* = 0.003), and diet quality (*p* = 0.03) were seen in the intervention group. There was also greater satisfaction at baseline (*p* = 0.04) and higher acceptability scores for all questions at the review appointments [4].

There were two phases to the HERALD study. The pre‐intervention phase represented usual care and the intervention phase represents the revised model where there was introduction of a structured, health literacy‐sensitive communication intervention. The comparison was undertaken to explore potential changes in communication behaviours attributable to the intervention, rather than to assess causal effects. Only participants with paired recordings (i.e., a new and a review appointment) were included in this study to enable within‐participant comparison of communication quality across timepoints and to reduce inter‐individual variability. All audio recordings of the consultation were undertaken with verbal and written permission from the patient and dietitian. All appointments in the public hospital outpatient setting for this study were booked for 60 min for new patients and 30 min for review patients. This timing is at the discretion of the dietitian. Ethical approval for this secondary analysis was obtained from the University of [name removed for peer review Health and Medical Human Research Ethics Committee (approval number 2019/ETH03723, date of approval 10 December 2018). Data was collected between February 2019 and March 2020 (with the study ceased due to the COVID19 lockdown). Verbal and written informed consent was obtained from all patient and dietitians prior to participating in the HERALD study. All digital audio recordings and data analysis spreadsheets were uploaded and saved in a de‐identified format to an online cloud storage system.

To assess the technical quality of the communication skills of dietitians, two researchers independently completed the 20 item DIET‐COMMS tool while simultaneously listening to the audio recordings. The DIET‐COMMS tool is a valid and reliable tool for measuring communication skills of dietitians in patient consultations [7, 10]. Self‐training was undertaken prior to completing assessments by both researchers (J. M., K. L.) to ensure the DIET‐COMMS tool was applied correctly [11]. For this study, item 18 of the DIET‐COMMS relating to assessment of non‐verbal communication was deliberately not evaluated as video recordings have been perceived as obtrusive, and negatively influence the behaviour of HCPs and patients [12]. Thus the total DIET‐COMMS score was calculated from the sum of the remaining 19‐items. If any query arose during the scoring of the DIET‐COMMS tool, the audio recording was paused and this item revisited. One researcher (J. M., a final year dietetic student) completed the DIET‐COMMS tool for all appointments. To ensure reliability in the scoring of the DIET‐COMMS tool, a second researcher and experienced renal dietitian and researcher (K. L.) applied the tool to a random sample equivalent to 15% of the original study sample size.

Each of the 19 DIET‐COMMS items were rated according to the tool instructions, applying a discrete scoring system with 0 (not done or not achieved), 1 (partly achieved or attempted) and 2 (fully achieved) [7]. A total score ranging between 0 and 38 was obtained for each appointment by summing scores from the 19 domains. If an item was not completed due to external factors the item was removed from the final score. The final score was converted to a percentage, and the communication skills of dietitian was classified as poor if scored between 0% and 40%, average if between 41%–75% and good/adequate if between 76% and 100% [7, 13].

The three categories for the global assessment using the DIET‐COMMS tool are a clear pass, borderline or fail. This requires five mandatory questions of the 19 elements of communication to be fully achieved in order to be rated as adequate overall. These items are shown in Table 1 and are described by the literature as essential for patient‐centred care [7, 12, 14, 15, 16, 17, 18, 19]. The global assessment score recognises and allows for variations in dietetic practice style. For this study, additional evidence for appointments that did not score a clear pass was documented in the form of field notes and used to discuss and achieve consensus when required.

**Table 1:** Mandatory items to achieve clear pass in the DIET‐COMMS tool [7].

Item number	Description
5	Establishes rapport
6	Checks understanding of medical condition
7	Offers information on how food relates to the condition
9	Works in partnership with client to identify possible dietary changes. Explores possible difficulties
11	Offers written information to reinforce verbal

The definition of high‐quality communication for this study was defined as a DIET‐COMMS score of > 75% [7] and a score of 10/10 for the five mandatory items. If a consultation achieved a score of > 75% but did not gain a maximum score of 10 for the mandatory items, they were categorised as not exhibiting high‐quality effective communication. Due to the inability to observe non‐verbal communication, item number 15 of the DIETCOMMS relating to active listening was assessed by listening for cues (‘yes’, ‘mm’, ‘I see’, ‘go on’, okay’) as these allow for patients to feel heard and encourage them to continue speaking [14]. Other cues for active listening such as paraphrasing the patient conversation, and use of open questions were used to indicate active listening and scored as fully demonstrated.

To determine the elements of a dietetic appointment that may change in response to a health literacy communication intervention, items in the DIET‐COMMS tool were grouped into six categories representing phases of the dietetic appointment. These phases were adapted from the Calgary‐Cambridge guide to the medical interview [20] as this was utilised in the development of the DIET‐COMMS tool [7]. These categories were: initiating the session, history taking, rapport building, counselling technique and active listening, closing the session and interview structure.

Information on participants were collected from the electronic medical record as part of routine care and included age, gender, presence of a carer in the appointment, anthropometry, biochemistry and reason for referral. Dietetic level of expertise was defined as either competent, proficient or expert according to the adapted Dreyfus model of skill acquisition [21].

### 2.1 Statistical Analysis

Scores for the DIET‐COMMS assessment were entered into Microsoft Excel v 16.36.

Statistical analysis was conducted in SPSS Software Version 26 (IBM Corp. Released 2019. Armonk, NY). Descriptive statistics were used to describe participant demographic and clinical data. Normality of data was determined using the Shapiro–Wilk test. Data is reported as mean and standard deviation or median and interquartile ranges. Categorical data is reported as counts and percentages (%) and the association between categorical variables was evaluated using Chi‐square test. Independent sample t‐tests or Wilcoxon rank sum test were used to determine if there were differences in DIET‐COMMS score or consultation time between the study periods. Inter‐rater reliability was assessed using Cohen's kappa to quantify agreement between evaluators beyond chance. Kappa values were interpreted according to established benchmarks (e.g., < 0.20 = poor, 0.21–0.40 = fair, 0.41–0.60 = moderate, 0.61–0.80 = substantial, > 0.80 = almost perfect agreement between raters) [22]. To ensure consistency across raters, all evaluators underwent standardised training using the DIETCOMMS tool prior to data collection; a subset of cases (15%) was independently scored by a second rater (KL); discrepancies were resolved through discussion. The resulting kappa value was 0.82, indicating excellent agreement. Statistical significance was set at a *p*‐value of < 0.05.

## 3 Results

**Figure 1:**
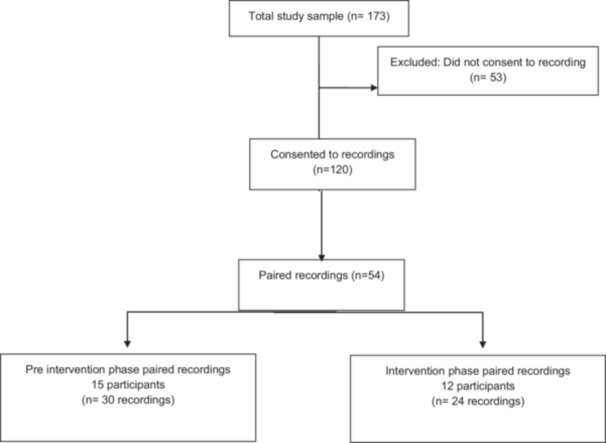
Study sample and flow.

Of the 27 participants with paired recordings, 15 attended the dietetic clinic in the pre‐intervention study phase and 12 in the intervention phase of the HERALD study [4]. The study flow is shown in Figure 1. The characteristics of the patient participants are shown in Table 2. The mean age was 65.5 ± 17.0 years, and 59.3% were male. Participants in the intervention period were significantly younger than those in the pre‐intervention period (61.5 ± 20.7 years vs. 68.8 ± 12.7, *p* < 0.001, Table 2). There was a significantly greater number of consults with a carer present in the intervention period than in the pre‐intervention period (66.7% vs. 20.0%, *p* < 0.001). Of the 54 appointments, 31 were conducted by dietitians considered as experts (57.4%) and 23 as proficient (42.6%). There was no significant difference in level of expertise between the two study periods (*p* = 0.07). The main reason for referral was pre‐dialysis education in the pre intervention phase (36.7%) and weight loss (38.1%) in the intervention phase.

**Table 2:** Participant demographic and clinical variables (*n* = 27).

	Pre‐Intervention *n* = 15 Mean ± SD	Intervention period *n* = 12 Mean ± SD	Total *n* = 27 Mean ± SD	*p*‐value
Age, years	68.8 ± 12.7	61.5 ± 20.7	65.5 ± 17.0	< 0.001
Gender (male) (*n*,%)	8 (53.3%)	8 (66.7%)	16 (59.3%)	0.32
Carer present (*n*,%)	6 (20.0%)	16 (66.7%)	22 (40.7%)	< 0.001
Cultural background Non‐CALD (*n*,%)	14 (93.3%)	11 (91.7%)	25 (92.6%)	1.00
Weight, kg	98.4 ± 25.9	81.5 ± 17.0	90.9 ± 23.8	0.10
BMI, kg/m^2^	35.7 ± 9.8	29.1 ± 6.8	32.7 ± 9.1	0.03
Potassium, mmol/L	4.80 ± 0.70	4.60 ± 0.60	4.70 ± 0.60	0.07
Phosphate, mmol//L	1.40 ± 0.30	1.20 ± 0.31	1.30 ± 0.20	0.31
eGFR mL/min/1.73 m^2^	27.9 ± 19.8	45.9 ± 31.3	35.9 ± 26.9	< 0.001
Reason for referral *n* (%)				
Weight loss	6 (20.0%)	8 (38.1%)	14 (25.9%)	0.63
Healthy eating	3 (10.0%)	3 (12.5%)	6 (11.1%)	
Low electrolyte diet	4 (13.3%)	2 (8.3%)	6 (11.1%)	
Pre dialysis	11 (36.7%)	5 (20.8%)	16 (29.6%)	
Miscellaneous	6 (20.0%)	6 (25.0%)	12 (22.2%)	
Appointments categorised according to dietitian level of expertise *n* (%)				
Proficient	16 (53.3%)	7 (29.1%)	23 (42.6%)	0.07
Expert	14 (46.7%)	17 (70.8%)	31 (57.4%)	
Review	12/15 (80.0%)	9/12 (75.0%)	21/27 (77.8%)	
Total	25/30 (83.3%)	19/24 (79.2%)	44/54 (81.5%)	
Consultation time (minutes) (median, Interquartile range, IQR)				
New	113 (IQR: 90–120)	90.0 (88.8–120)	100 (90–120)	0.22
Review	75 (54–90)	60 (45–76)	60 (45–90)	
Total	90 (60–120)	80 (60–90)	90 (60–105)	

*Note:* Additional information on level of expertise is shown in Supporting materials Table 1.

Abbreviations: CALD, culturally and linguistically diverse background; QPS, question prompt sheet; SD, standard deviation.

### 3.1 Technical Quality of Dietetic Communication Skills

Overall, the mean DIET‐COMMS score for all appointments was 80.9 ± 10.2% indicating high quality communication (Table 3). This did not differ according to study phase, appointment type or level of dietetic expertise. When scores were dichotomised as high quality or clear passes, there was also no difference in the proportion according to study phase, appointment type or level of dietetic expertise.

**Table 3:** DIET‐COMMS scores for study participants (*n* = 27).

	Pre‐Intervention *n* = 15 Mean ± SD	Intervention period *n* = 12 Mean ± SD	Total *n* = 27 Mean ± SD	*p*‐value
DIET‐COMMS score according to appointment type (mean ± SD, %)				
Initial appointment	83.0 ± 7.0	82.4 ± 11.1	82.7 ± 8.9	0.10
Review appointment	79.4 ± 9.5	78.4 ± 12.7	79.0 ± 11.3	0.68
All appointments	81.2 ± 9.0	80.4 ± 11.8	80.9 ± 10.2	0.22
DIET‐COMMS score according to dietetic experience level (mean ± SD, %)				
Proficient	77.6 ± 9.7	76.7 ± 9.8	79.0 ± 11.3%	0.46
Expert	84.8 ± 6.8	84.3 ± 9.8	84.5 ± 8.42%	0.20
Proportion of high quality dietetic appointments according to appointment type (n,%)				
Initial	13/15 (86.7%)	10/12 (83.3%)	23/27 (82.2%)	0.97
Review	12/15 (80.0%)	9/12 (75.0%)	21/27 (77.8%)	
Total	25/30 (83.3%)	19/24 (79.2%)	44/54 (81.5%)	
Proportion of clear passes according to appointment type (n,%)				
Initial	11/15 (73.3%)	6/12 (50.0%)	17/27 (63.0%)	1.00
Review	11/15 (73.3%)	6/12 (50.0%)	17/27 (63.0%)	
Total	22/30 (73.3%)	12/24 (50.0%)	34/54(63.0%)	
Proportion of high quality dietetic appointments according to level of dietetic expertise (*n*,%)				
Proficient	9/16 (56.3%)	0/7(0.00%)	9/23 (39.1%)	0.09
Expert	13/14 (92.9%)	12/17 (70.6%)	25/31 (80.6%)	
DIET‐COMMS score according to intervention component used (mean ± SD, %)				
Score all appointments	81.2 ± 9.0	80.4 ± 11.8	80.9 ± 10.2	0.22
QPS only (*n* = 4)	n/a	73.3 ± 8.1	n/a	0.18
Teachback only (*n* = 9)	n/a	79.0 ± 14.9	n/a	
QPS + teachback (*n* = 4)	n/a	86.0 ± 6.08	n/a	
Nil used (*n* = 7)	n/a	75.6 ± 7.11	n/a	

Abbreviations: n/a, Not applicable; QPS, question prompt sheet; SD, standard deviation.

There was no significant difference in DIET‐COMMS score when specific elements of the intervention were examined (Table 3). However, use of teachback and the QPS showed a higher overall DIET‐COMMS score (86.0 ± 6.1%) compared to QPS only (73.3 ± 8.1%) or use of neither (75.6 ± 7.1%).

### 3.2 Changes in Dietetic Communication Skills as a Result of a Communication Intervention

To evaluate which facets of communication may change in response to a communication intervention, the mean DIET‐COMMS score for each of 19 DIET‐COMMS item number and the six phases of a dietetic outpatient appointment were examined. Mean scores are shown in Table 4 and indicate that there were significant changes in mean scores between pre intervention and intervention period. This included a decrease in score for Item 8 (completes assessment [clinical, behavioural, dietary]) (Table 4, mean score pre‐ intervention 1.97 compared to 1.88 in the intervention period, *p* = 0.01); and a decrease in Item 15 (active listening) (mean score pre intervention 2.00 compared to 1.83 in intervention period, *p* = < 0.001, Table 4). There was also a decrease overall in the interview structure phase of the appointment including reductions in item 13 (logical sequence) (Table 4, mean score pre intervention 1.93 compared to 1.83 intervention period, *p* = 0.02) and item 14 (completed in timely manner) (mean score pre intervention 1.90 compared to 1.67 intervention, *p* = < 0.001). In contrast, there was a significant increase in scores for Item 7 (offers information on how food relates to condition) mean score 1.77 pre and 1.88 intervention period, *p* = 0.04 (Table 4).

**Table 4:** DIET‐COMMS item scores according to study period.

	Pre‐Intervention *n* = 15 Mean ± SD	Intervention period *n* = 12 Mean ± SD	Total *n* = 27 Mean ± SD	*p*‐value
*Initiating the session. Maximum score: 2*				
Initiating the session.	1.50 ± 0.71	2.00 ± 0.00	1.88 ± 0.35	0.08
1 Greeting and introduction	1.50 ± 0.71	2.00 ± 0.00	1.88 ± 0.35	0.08
*History taking. Maximum: 8*				
History taking	6.20 ± 1.30	6.29 ± 1.37	6.24 ± 1.33	0.77
2 Clarifies reason for referral	1.57 ± 0.57	1.58 ± 0.58	1.57 ± 0.57	0.99
3 Outlines appointment	0.87 ± 0.90	1.08 ± 0.88	0.96 ± 0.89	0.67
4 Listen/Understanding	1.80 ± 0.41	1.75 ± 0.44	1.78 ± 0.42	0.40
8 Dietary assessment	1.97 ± 0.34	1.88 ± 0.34	1.93 ± 0.26	0.01
*Rapport building. Maximum score: 8*				
Rapport building	6.27 ± 1.64	5.83 ± 1.90	6.07 ± 1.76	0.37
5 Establishes rapport	1.47 ± 0.63	1.63 ± 0.65	1.54 ± 0.64	0.65
6 Checks understanding	1.53 ± 0.68	1.17 ± 0.82	1.37 ± 0.76	0.30
16 Non judgemental	1.63 ± 0.49	1.63 ± 0.58	1.63 ± 0.53	0.52
17 Acknowledges feelings	1.63 ± 0.49	1.48 ± 0.65	1.54 ± 0.57	0.06
*Counselling technique and active listening. Maximum score: 10*				
Counselling technique and active listening	8.40 ± 1.25	8.04 ± 2.24	8.24 ± 1.75	0.11
7 Information provided	1.77 ± 0.43	1.88 ± 0.34	1.81 ± 0.39	0.04
9 Partnership and explores	1.67 ± 0.48	1.58 ± 0.58	1.63 ± 0.53	0.17
10 Plan with key goals	1.48 ± 0.58	1.54 ± 0.71	1.51 ± 0.64	0.36
12 Agrees next step	1.63 ± 0.56	1.54 ± 0.59	1.59 ± 0.57	0.48
15 Uses active listening	2.00 ± 0.00	1.83 ± 0.38	1.93 ± 0.26	< 0.001
*Closing the session. Maximum score: 6*				
Closing the session	4.77 ± 0.97	4.96 ± 1.08	4.85 ± 1.02	0.48
11 Written information	1.66 ± 0.77	1.71 ± 0.69	1.68 ± 0.73	0.54
19 Appropriate language	1.57 ± 0.57	1.67 ± 0.56	1.61 ± 0.56	0.49
20 Summarises	1.60 ± 0.56	1.58 ± 0.58	1.59 ± 0.57	0.83
*Interview structure. Maximum score: 4*				
Interview structure	3.83 ± 0.38	3.50 ± 0.66	3.69 ± 0.54	< 0.001
13 Logical sequence	1.93 ± 0.25	1.83 ± 0.38	1.89 ± 0.32	0.02
14 Timely fashion	1.90 ± 0.32	1.67 ± 0.57	1.80 ± 0.45	< 0.001

Abbreviation: SD, standard deviation.

## 4 Discussion

This study aimed to evaluate the technical quality of communication skills demonstrated by dietitians in an outpatient nephrology clinic setting and to explore how these skills may be influenced by a structured communication intervention. The findings were that the majority of appointments (81.5%) demonstrated effective communication, with a mean DIET‐COMMS score of 80.9%. This is consistent with the findings of several other studies that found that dietitians are generally exhibiting effective communication [7, 8, 23, 24]. However, previous literature is mostly qualitative in nature [7, 8, 24, 25] and this present finding extends the current evidence base on the communications skills of dietitians by providing quantitative data on the technical quality of these skills. The only other comparable study to date that has used the DIET‐COMMS tool was in the inpatient setting and found that 85‐98% of dietitians scored > 75% on the DIET‐COMMS tool [8]. However, the slightly lower scores observed in this study may reflect the absence of extensive pre‐intervention training, which has been shown to enhance performance in similar contexts [8].

Our findings exceed the scores obtained using other quantitative tools or observations in medical appointments. In these studies doctors typically demonstrated effective communication between 40% and 76% of the time [26, 27]. Interestingly, this study found no significant differences in communication quality based on appointment type (initial vs. review) or dietitian experience level (proficient vs. expert). This suggests a degree of consistency in communication practices across clinical contexts despite the variation in appointment structure [7, 27, 28]. This finding also aligns with findings from Hancock et al. [14], who reported that consistent use of effective communication strategies positively influenced patients’ perceptions of care quality. However, this consistency masks underlying variability in specific communication domains, particularly those most affected by the intervention [29].

A key and somewhat unexpected finding was the decline in active listening following the implementation of the communication intervention. While literature on active listening in dietetics is limited, recent studies suggest that dietitians often perceive their training in this area as inadequate [30]. A 2023 scoping review by Knight et al. [3] argue for further development of communication skills commencing in University and continuing throughout professional practice to strengthen the technical quality of dietitians communication skills. This assists those who are novices to strengthen newly acquired skills and enhances more proficient staff who may need ongoing practice and reinforcement as communication and counselling skills are known to be enhanced over time via the use of appropriate tools and feedback [31, 32]. This is also underscored by findings that around 40% of dietitians feel their tertiary education did not adequately prepare them in communication and active listening skills [25, 33]. This is concerning given that active listening is a cornerstone of patient‐centred care and has been linked to improved patient adherence, satisfaction, and appointment attendance [34, 35, 36, 37, 38, 39].

The observed decline in active listening may be attributed to the cognitive load introduced by the intervention, particularly the use of the renal diet specific QPS [40]. Audio recordings indicated that patients often initiated discussions based on the QPS very early in the consultation, which disrupted the traditional sequencing of the Nutrition Care Process. This shift in structure may have inadvertently reduced opportunities for dietitians to engage in reflective listening, as they prioritised responding to patient questions. This finding aligns with Clayton et al. [41], who reported that QPS use in oncology also altered the consultation dynamic and distribution of time across consultation components.

Despite these challenges, the QPS was associated with increased patient engagement and a higher volume of patient‐initiated questions [4], consistent with findings from Lambert et al. [9], who demonstrated that a renal diet‐specific QPS significantly improved the patient‐centeredness of dietetic consultations without increasing the overall volume of communication. This suggests that while a QPS may disrupt traditional consultation flow, it can enhance the relevance and responsiveness of communication from the patient's perspective. This may have also contributed to the findings in the main study of greater satisfaction and acceptability [4].

Another promising but underpowered finding was that appointments incorporating both the QPS and teachback method achieved the highest mean DIET‐COMMS scores. Although the sample size was small, this aligns with a growing body of evidence supporting the effectiveness of teachback in improving patient understanding, recall and adherence. Teachback has been reported to be of benefit to patients with low health literacy [42], which is common in CKD [43, 44]. Furthermore, teachback can assist patients and carers to make sense of dietary advice [2, 45]. However, it must be noted that the practice of teachback may not be appropriate for every patient (such as those with cognitive impairment [46, 47, 48]) and based on the pragmatic nature of the HERALD study [4] it was not incorporated into every appointment. Despite this, numerous systematic reviews have found that teachback improves patient self‐efficacy and adherence to recommended health behaviours across a range of clinical settings [49, 50, 51].

This research is limited by utilising a small sample of patients from a single local health district, and an unequal number of participants between study groups and may not be a true representation of all nephrology dietetic appointments. Although blinded to their scores, dietitians may have attempted to self‐improve their communication skills or style. Additionally, the findings of this study cannot be generalised to the other dietetics specialty areas or healthcare contexts.

Another limitation is the small sample of dietitians utilised in this study (*n* = 7), which may have created a cluster effect causing the results to be unrepresentative of all dietitians. Furthermore, the utilisation of video‐recorded appointments for analysis would have allowed for a greater exploration of non‐verbal communication skills. While non‐verbal communication is a vital part of communication process, the broader literature suggests that video recording may be perceived as obtrusive, and negatively influence the behaviour of HCPs and patients [52, 53]. In addition video‐recordings may result in feelings of intimidation or social anxiety among dietitians [7]. It must be noted that the communication skills of dietitians in the present study may also have been altered by an awareness that they were being recorded, therefore potentially resulting in an observer and social desirability effect. While active listening was measured in this study, exploring non‐verbal communication would have enriched findings. Finally, all of the dietitians conducting appointments were female and research suggests that gender bias can influence the appointment [54, 55].

Based on the pragmatic nature of this study, not all dietetic appointments included the intervention components and therefore the true measure of effect was not able to be determined. It may be premature to make statistical inferences as this study used multiple *p* value testing and had no power calculation data for the effect of communication interventions in dietetics or the DIETCOMMS. Future studies can use these findings to conduct adequately powered trials. Finally, qualitative interviews with patients about the intervention and potential effects on behaviour change were planned but unable to be conducted due to commencement of lockdowns due to the COVID 19 pandemic and redirection of staff to other activities.

While there are limitations to this study, there are several strengths. Firstly, it is the second known study to implement the DIET‐COMMS tool in the clinical setting and provides useful data on the need for additional training in communication skills. Secondly, the pragmatic nature of participant recruitment in the HERALD study [4], allowed the researchers to make assessments of the technical quality of dietetic communication skills in a complex cohort of patients with multiple co‐morbidities, low levels of health literacy and cognitive impairment [43, 46]. To this end, the findings may be utilised to inform renal dietitians about several facets of communication that may enhance the patient centredness of renal care. This includes the identification of specific domains impacted by a communication intervention (such as active listening and the interview structure). Finally, the exploration of communication skills in real dietetic outpatient appointments rather than simulated appointments strengthen the findings of this study and its application to dietetic practice. Nevertheless, studies involving wider groups of dietetic professionals in addition to a range of clinical areas and contexts such as the inpatient setting are needed to explore the communication skills of dietitians in more detail, to ascertain the findings of this study.

## 5 Conclusion

This study assessed the technical quality of dietetic communication in renal outpatient consultations and examined changes following a targeted communication intervention. Across initial and review appointments, 81.5% of consultations demonstrated effective communication (mean DIET‐COMMS score: 80.9%, range: 61%–100%), with no significant differences by appointment type, intervention period, or dietitian experience level. Although the intervention did not significantly enhance overall communication scores from baseline, it identified key areas that were altered as a result of the intervention, including conducting the assessment, active listening and appointment structure and timing. These findings highlight the need for strategies that strengthen communication without extending consultation duration. Future research should explore scalable interventions, including the use of teachback across diverse clinical contexts, to evaluate their impact on patient outcomes. The results support the continued use of the DIET‐COMMS tool to quantify communication quality and guide professional development across dietetic specialties. This study also contributes to the limited evidence base on dietetic communication and underscores the importance of targeted approaches to enhance nutrition counselling in outpatient care.

## Author Contributions

**Jenna Mazabow:** conceptualization (supporting), investigation, formal analysis (lead), writing – original draft (lead). **Denelle Cosier:** formal analysis, writing – review and editing. **Kelly Lambert:** conceptualization, funding acquisition, investigation, writing – review and editing (lead), supervision, project administration.

## Ethics Statement

Approval received from the University of Wollongong Health and Medical Human Research Ethics Committee (2019/ETH03723).

## Conflicts of Interest

The authors declare no conflicts of interest.

## Supporting information

Supporting File:

## Data Availability

The data that support the findings of this study are available on request from the corresponding author. The data are not publicly available due to privacy or ethical restrictions.
