# Supplementary material for: Evaluating the Impact of a Health Literacy Intervention on the Quality of Dietetic Communication in Outpatient Kidney Care
Source: J Hum Nutr Diet. 2026 Jun 25;39(3):e70296. doi: 10.1111/jhn.70296 (PMC13304251; doi:10.1111/jhn.70296)
Supplement: Supplementary file 1 — Supporting File: [file JHN-39-0-s001.docx]

Supplementary materials

Supplementary Table 1

Participant demographic and clinical variables (n=27)

|  | Pre-Intervention  n=15  Mean ±SD | Intervention period  n=12  Mean ±SD | Total  n=27  Mean ±SD | P-value |
| --- | --- | --- | --- | --- |
| New appointment | | | | 0.33 |
| Expert | 5 | 2 |  |  |
| Proficient | 12 | 8 |  |  |
| Novice | 15 | 2 |  |  |
| Review appointment | | | |  |
| Expert | 11 | 7 |  |  |
| Proficient | 6 | 3 |  |  |
| Novice | 11 | 2 |  |  |
|  |  |  |  |  |
